# The Excitatory Synaptic Transmission of the Nucleus of Solitary Tract Was Potentiated by Chronic Myocardial Infarction in Rats

**DOI:** 10.1371/journal.pone.0118827

**Published:** 2015-03-10

**Authors:** Jing Li, Ming-Ming Zhang, Ke Tu, Jian Wang, Ban Feng, Zi-Nan Zhang, Jie Lei, Yun-Qing Li, Jian-Qing Du, Tao Chen

**Affiliations:** 1 Department of Physiology and Pathophysiology, Xi’an Jiaotong University, School of Medicine, Xi’an, 710061, China; 2 Department of Anatomy, Histology and Embryology, K. K. Leung Brain Research Centre, Fourth Military Medical University, Xi’an, 710032, China; 3 Center for Neuron and Disease, Frontier Institute of Science and Technology, Xi’an Jiaotong University, Xi’an, China; Xi’an Jiaotong University School of Medicine, CHINA

## Abstract

Angina pectoris is a common clinical symptom that often results from myocardial infarction. One typical characteristic of angina pectoris is that the pain does not match the severity of the myocardial ischemia. One possible explanation is that the intensity of cardiac nociceptive information could be dynamically regulated by certain brain areas. As an important nucleus for processing cardiac nociception, the nucleus of the solitary tract (NTS) has been studied to some extent. However, until now, the morphological and functional involvement of the NTS in chronic myocardial infarction (CMI) has remained unknown. In the present study, by exploring left anterior descending coronary artery ligation surgery, we found that the number of synaptophysin-immunoreactive puncta and Fos-immunoreactive neurons in the rat NTS two weeks after ligation surgery increased significantly. Excitatory pre- and postsynaptic transmission was potentiated. A bath application of a Ca^2+^ channel inhibitor GABApentin and Ca^2+^ permeable AMPA receptor antagonist NASPM could reverse the potentiated pre- and postsynaptic transmission, respectively. Meanwhile, rats with CMI showed significantly increased visceral pain behaviors. Microinjection of GABApentin or NASPM into the NTS decreased the CMI-induced visceral pain behaviors. In sum, our results suggest that the NTS is an important area for the process of cardiac afference in chronic myocardial infarction condition.

## Introduction

Angina pectoris is a common clinical symptom that often results from ischemia of the heart muscle due to the occlusion of the coronary artery [1]. The typical manifestation of angina pectoris is often described as chest pain that is characterized by a retrosternal crushing, burning or squeezing sensation. Normally, the pain is continuous, deep and diffuse. However, one typical characteristic of angina pectoris is that the chest pain is not consistent with the development or severity of the myocardial ischemia: patients with severe myocardial ischemia may feel weak or no angina pectoris, while others with slight coronary artery occlusion may suffer from serious angina pectoris [1,2]. It is now accepted that one of the main reasons for the poor correlation between the symptoms and extent of the myocardial ischemia is the varying central sensitization of certain brain areas that are responsible for the processing and regulation of visceral nociceptive information [2,3,4]. However, in comparison with the widely studied central sensitization for somatic nociceptive information, our understanding of central modulation for visceral information lags far behind.

The nucleus of the solitary tract (NTS), located in the medulla oblongata, is an important nucleus for processing cardiac nociception. Nociceptive information from the heart can be directly forwarded to the NTS through the afferent fibers of the vagal nerve. In addition, sympathetic afferent fibers can transmit cardiac nociceptive information to the dorsal horn of the spinal cord and then to the NTS through ascending pathways [1,2,5,6,7]. Pericardial application of algogenic compounds (e.g., adenosine, capsaicin, bradykinin) or temporary occlusion of the left coronary artery significantly increases c-Fos expression in the NTS, suggesting that the NTS is important for the central regulation of angina pectoris after a heart attack [8,9]. In our previous work, we also found that the NTS was involved in the facilitation of the acute cardiac-somatic reflex. NMDA receptors and group III mGluRs play different roles in mediating this facilitation effect [10,11]. However, up to now, the role of the NTS in angina pectoris has only been studied in acute heart attack models. To date, no data have shown the morphological and functional involvement of the NTS in a chronic myocardial ischemia model.

In the present study, by exploring left anterior descending coronary artery ligation surgery, we thus explored the morphological and functional changes of the NTS in the presence of chronic myocardial infarction (CMI) in rats. The possible visceral pain behavior changes were also tested.

## Materials and Methods

### Animals

Male adult SD rats (8–10 weeks old) were used. The animals were randomly housed under a 12-h light-dark cycle, with food and water freely available, at least one week before use. The experiments were carried out in a blind manner, in which the drug preparation, experiments and data analyses were performed by different people. Animal experiments were performed according to the ethical guidelines of the International Association for the Study of Pain and approval from the Animal Use and Care Committee for Research and Education of the Fourth Military Medical University (Xi’an, China) and Xi’an Jiaotong University. All efforts were made to minimize animal suffering and the number of animals used.

### Left anterior descending coronary artery ligation

Left anterior descending coronary artery ligation was performed to produce a CMI rat model, using techniques described in detail elsewhere [9]. Briefly, in isoflurane-anesthetized rats, 2-cm incisions were made to the left of and parallel to the sterni. Then, the fifth and sixth ribs were separated with a clamp, and the hearts were exteriorized by applying pressure to the lateral aspects of the thoracic cage. The left descending coronary arteries were then occluded 1 to 2 mm from their origins with a 6–0 prolene suture. The chests were closed, and the rats were allowed to recover for two weeks before the following experiments. Sham-operated rats underwent an identical surgery but did not sustain a left descending coronary artery ligation.

### Detection of myocardial enzymes

Blood from the femoral artery was used for cardiac enzyme detection with a myocardial enzyme kit (Nanjing Jiancheng Bioengineering Institute, China) one day after coronary artery ligation. The plasma levels of the cardiac enzymes L-lactate dehydrogenase (LDH-L), creatine kinase (CK) and creatine kinase MB isoenzyme (CK-MB) were determined by a 721-series visible spectrophotometer (manufactured by Shanghai Precision & Scientific Instrument Co., Ltd.; the UV-V is a spectrophotometer of the UV300 type).

### Immunohistochemical study

After recovering from surgery for 14 days, all rats were anesthetized with an overdose of sodium pentobarbital (100 mg/kg, i.p.) and perfused with 100 ml of 0.9% saline, followed by 500 ml of 0.1 M phosphate buffer (PB, pH 7.4) containing 4% (w/v) paraformaldehyde. After perfusion, the heart tissue containing the left ventricle and the brainstem containing the NTS were removed immediately and placed in cold 4% paraformaldehyde in 0.1 M PB for 2 hr. The heart tissue and brainstem were then saturated with 30% (w/v) sucrose in 0.1 M PB overnight at 4°C and cut into 30 μm-thick serial frontal sections on a freezing microtome (Kryostat 1720; Leitz, Mannheim, Germany). The sections were collected serially into 4 dishes containing 0.05 M phosphate-buffered saline (PBS, pH 7.4) as 4 sets of every fourth serial section. Each dish contained a complete set of the serial sections and was incubated in 0.01 mol/L Tris-HCl buffered saline with 2% normal goat serum for half an hour. The heart sections were processed with conventional HE staining, and the brainstem sections were processed with immunostaining as following:

The first set of sections from the brainstem containing NTS was used for Fos immunostaining. Sections were sequentially incubated with rabbit anti-Fos antiserum and biotinylated anti-rabbit IgG in PBS containing 5% (v/v) NDS, 0.3% (v/v) Triton X-100 and 0.05% (w/v) NaN3 (PBS-NDS) for 12–24 hr at room temperature. Sections were then incubated in avidin-biotin complex (ABC Solution, Vector Laboratories. Inc. USA) for 1 hr and reacted with 0.04% diaminobenzidinetetrahydrochloride (DAB) (Dojin, Kumamoto, Japan) and 0.003% H_2_O_2_ for the visualization of Fos-immunoreactive (ir) neurons. Finally, sections were mounted on glass slides, air-dried, dehydrated, cleared and coverslipped for light microscopic visual examination (AHBT3; Olympus, Tokyo, Japan).

The second set of sections from the brainstem containing NTS was used for triple immunofluorescence staining. Sections were incubated with rabbit anti-Fos antiserum, mouse anti-synaptophysin antiserum and chicken anti-NeuN antiserum for 48 hr at 4°C. The sections were then incubated with Alexa 594-conjugated donkey anti-rabbit IgG, Alexa 488-conjugated donkey anti-mouse IgG and biotinylated anti-chicken IgG for 12 hr at room temperature, followed by incubation with Alexa 647-conjugated avidin. Finally, sections were mounted onto glass slides, air-dried and observed under a confocal laser scanning microscope (FV1000; Olympus, Tokyo, Japan).

The third set of serial brainstem sections was processed for Nissl staining. The fourth set of serial brainstem sections was used as a control. In the control experiments, the primary antibodies were omitted or replaced with a mixture of normal rabbit, mouse and chicken sera, and the remaining steps were performed identically to the procedure for the sections from the third dish. In the control experiments, no immunostaining product was detected.

### Antibodies

Antibody against Fos (Ab102699, 1:500) was purchased from Abcam, USA. Antibody against NeuN (70R-10629, 1:200) was purchased from Fitzgerald, Inc, USA. Antibodies against synaptophysin (MAB368, 1:200) and biotinylated anti-rabbit IgG (AP182B, 1:200) were purchased from Millipore Corporation, USA. Biotinylated anti-chicken IgG (BA-9010, 1:200) and an ABC kit (PK-4001) were purchased from Vector Laboratories, Inc, USA. Alexa 594-conjugated donkey anti-rabbit IgG (A21207, 1:500), Alexa 488-conjugated donkey anti-mouse IgG (A21202, 1:500) and Alexa 647-conjugated avidin (S21374, 1:1000) were purchased from Invitrogen, Thermo Fisher Scientific, Inc, USA.

### Whole-cell patch recording

Rats were anesthetized with ether and decapitated. The entire brainstem containing the NTS was rapidly removed and transferred to an ice cold oxygenated solution containing (in mM) 252 Sucrose, 2.5 KCl, 0.5 CaCl_2_, 6 MgSO_4_, 26 NaHCO_3_, 1.2 NaH_2_PO_4_, and 10 glucose, pH 7.4, osmolality 310–320 mOsm. After cooling for approximately 1–2 min, the brainstem block was cut into coronal slices (300 μm) by a vibrating tissue slicer (Leica VT1200S). and then transferred to a chamber with oxygenated artificial cerebrospinal fluid (ACSF) containing (in mM) 124 NaCl, 2.5 KCl, 2 CaCl_2_, 1 MgSO_4_, 25 NaHCO_3_, 1 NaH_2_PO_4_, and 10 glucose at room temperature for at least 1 hr [12]. Experiments were then performed in a recording chamber superfused with ACSF at a flow rate of 4 ml/min (34°C). For recording in the NTS, evoked EPSCs were recorded from neurons in the SolM, and the stimuli were delivered by a concentric bipolar tungsten stimulating electrode placed in the adjacent solitary tract (approximately 1 mm away from the recorded neuronal soma). Paired stimuli at an interval of 50 ms was induced at 0.033 Hz, and neurons were voltage-clamped at-70 mV. The recording pipettes (3–5 MΩ) were filled with a solution containing (in mM): 112 Cs-Gluconate, 5 TEA-Cl, 3.7 NaCl, 0.2 EGTA, 10 HEPES, 2 MgATP, 0.3 Na3GTP and 5 QX-314 (adjusted to PH 7.2 with CsOH, 290 mOsmol). Additionally, 0.1 mM spermine was included in the solution when recording the AMPA I-V curve. The junction potential was about 10 mV and was corrected. The I–V analysis were recorded in the presence of the NMDA receptor antagonist AP5 (50 μM). Reversal potential (E_rev_) values were estimated from the I–V relationship by linear interpolation. To calculate the rectification index (RI), we used the equation described in [13]: RI = (I_+40_/(40 − E_rev_))/(I_−70_/(70 − E_rev_)), where I_+40_ and I_−70_ are the EPSC current amplitude measured at +40 mV and at −70 mV, respectively. Picrotoxin (100 μM) and strychnine (2 μM) were always present to block GABA_A_ and glycine receptor-mediated inhibitory synaptic current in all experiments. TTX (1 μM) was added to the ACSF for recording the miniature excitatory post-synaptic current (mEPSC). The initial access resistance was 15–20 MΩ and was monitored throughout the experiment. Data were discarded if the access resistance changed by > 15% during experiment. Data were filtered at 1 kHz and digitized at 10 kHz.

### Behavioral testing

#### Drug microinjection into the NTS

Saline, NASPM or GABApentin was microinjected into the commissural part of the nucleus of the solitary tract (SloC) to test their effects on visceral pain behavior. Two weeks after coronary artery ligation surgery, the rats were anesthetized with inhaled isofluorane (1–3%, or as needed) and mounted in a stereotaxic frame (Narishige, Tokyo, Japan). Then, 0.2 μl of saline (0.9%), NASPM (3 mM) or GABApentin (5 mM) solution was injected at a rate of 0.05 μl/min into the SolC (coordinates: 7.92 mm posterior to the bregma, along the midline and 4.30 mm ventral to the skull) through a glass micropipette (inner tip diameter, 15–20 μm) attached to a 1 μl Hamilton microsyringe. After injection, the surgical wound was subcutaneously injected with 2% lidocaine as a local anesthesia and allowed to recover from anesthetization for 45 min. The injection sites were confirmed at the end of all the experiments, and sites outside of the SolC region were excluded from the study.

#### Open field

Forty-five min after microinjection, rats were placed in an open field (100 × 100 × 40 cm^3^) inside a dimly lit isolation chamber (<50 lux in the center of the open field) with a fan. An activity monitoring system (Smart v 2.5.21, Panlab Inc. Spain) was used to record horizontal locomotor activity. Briefly, this system used paired sets of photo beams to detect movement in the open field and movement was recorded as beam breaks. Each animal was placed in the center of the open field, and vertical (rearing, rats stood up with hind limbs) activity and travel distance were measured for 15 min.

### Hemodynamic Studies

Two weeks after the surgery, rats were lightly anesthetized with ether. The right carotid artery was isolated by cut-down, and a 24 G arteriovenous indwelling needle (Bayan Lepas Free Inc. Malaysia) was passed retrogradely into the artery under constant pressure monitoring. An analog signal was passed through an electronic differentiator to record heart rate and mean arterial pressure.

### Statistical analysis

Statistical comparisons were made using unpaired and paired t-test (SigmaPlot 12.0; Systat Software, CA, USA). All data are presented as the Mean ± S.E.M. In all cases, *p* < 0.05 was considered statistically significant.

## Results

### Increased myocardial enzymes and damaged myocardial tissue after coronary artery ligation

**Fig 1:**
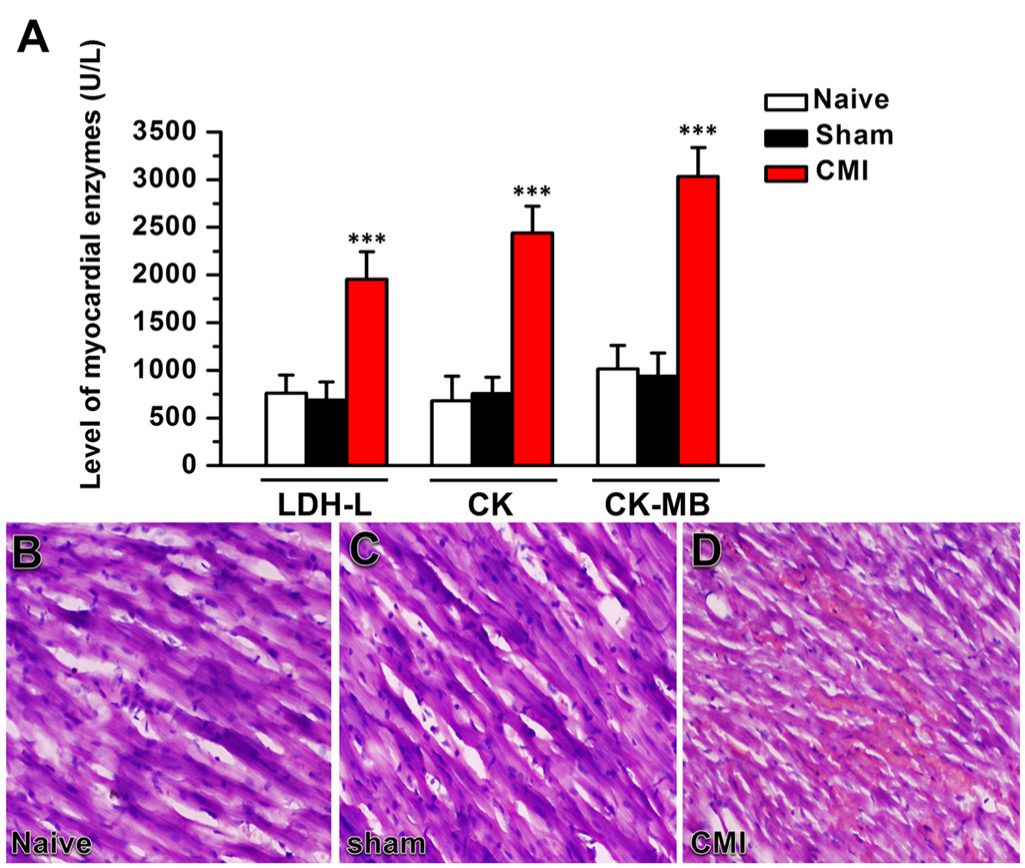
Coronary artery ligation increased the plasma level of myocardial enzymes and damaged myocardial tissue. (A) The plasma level of the myocardial enzymes LDH-L, CK and CK-MB (U/L) in naïve rats and rats with sham operation and CMI surgery. ***, *p* < 0.001 compared with the naïve and sham groups. (B-D) HE staining showing the myocardial tissue of the left ventricle in naïve rats (B), rats with the sham operation (C) and rats with CMI surgery (D). LDH-L, L-lactate dehydrogenase; CK, creatine kinase; CK-MB, creatine kinase MB isoenzyme; CMI, chronic myocardial infarction. Scale bar: 50 μm in (B-D).

To confirm that the left anterior descending coronary artery ligation surgery was successfully performed and the chronic myocardial infarction (CMI) model was properly established, we tested the level of plasma cardiac enzymes LDH-L, CK and CK-MB one day after the surgery [14] and checked the myocardial tissue of the left ventricle by HE staining 14 days after the surgery. The levels of LDH-L, CK and CK-MB were significantly increased in rats with coronary artery ligation compared with naïve and sham surgery rats (*p* < 0.001, n = 6 rats in naïve and sham group and 7 rats in CMI group, one-way ANOVA followed by *post hoc* comparison with LSD test) (Fig. 1A). The HE staining results showed no morphological damage in the myocardial tissue of the naïve and sham surgery rats. However, in rats with left anterior descending coronary artery ligation, moderate to severe cardiomyocytes damage was detected, showing sarcolemma breakage, subsarcolemmal cytoplasmic swelling and shrinking of the nuclear membrane. In addition, a mild hemocyte infiltration was observed in the myofibers (Fig. 1B-D). Based on these results, we believe that the CMI model was successfully established in the present study.

### The overexpression of Fos- and synaptophysin-immunoreactivities in the NTS after CMI

**Fig 2:**
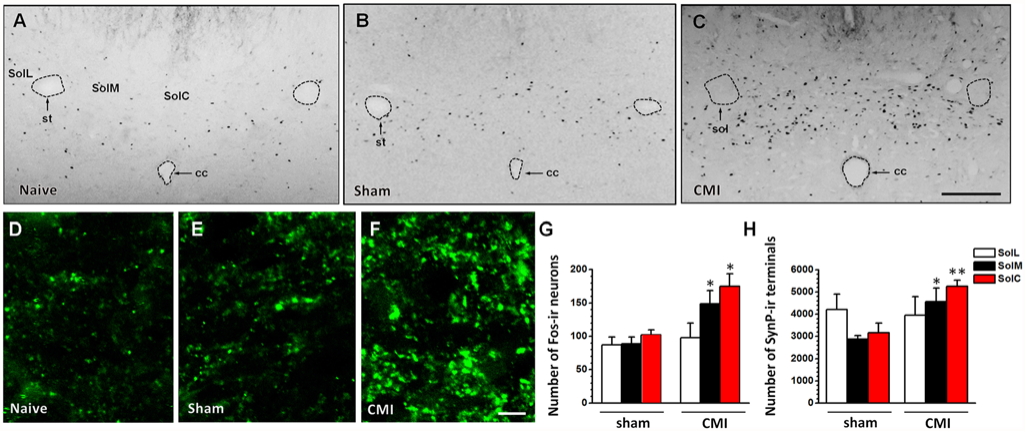
Immunostaining results showing the expression of Fos-ir neurons and synaptophysin-ir puncta in the NTS. The expression of Fos-ir neurons was not increased in rats with sham surgery but was increased significantly in rats with CMI, compared with naïve rats (A-C). Synaptophysin expression also increased in the CMI group compared with the naïve and sham groups (D-F). The summarized results for Fos-ir neurons and synaptophysin-ir puncta are plotted in (G-H). *, *p* < 0.05; **, *p* < 0.01 compared with the sham group. cc, central canal of the brainstem; CMI, chronic myocardial infarction; SolC, commissural part of the NTS; SolL, lateral part of the NTS; SolM, medial part of the NTS; st, solitary tract. Scale bars: 500 μm (A-C) and 10 μm (D-F).

Fos protein is a widely used molecular marker for showing neural activation after somatic or visceral stimuli [12,15,16,17]. We thus used Fos staining to determine whether the NTS is involved in the central regulation of CMI. The results showed that the sham surgery rats did not exhibit increased Fos expression compared with the naïve rats (n = 4 rats, p > 0.05, unpaired t-test) (Fig. 2A-B, G). However, in rats with CMI, significantly increased Fos expression was observed in the medial (SolM) and commissural (SolC) part of the NTS compared with rats in the sham group (SolM: sham, 89.0 ± 10.4, CMI, 149.3 ± 19.7, p = 0.03; SolC: sham, 101.8 ± 7.8, CMI, 176.0 ± 18.9, p = 0.01. n = 4 rats in each group, unpaired t-test). Furthermore, there was no difference in Fos expression in the lateral part of the NTS (SolL) between the CMI and sham groups (sham, 87.0 ± 12.0, CMI, 98.3 ± 21.8; p = 0.67, n = 4 rats in each group, unpaired t-test) (Fig. 2B-C, G).

We also measured the density of presynaptic boutons by using immunostaining for synaptophysin and analyzing the number of synaptophysin-immunoreactive (ir) puncta in the NTS [18]. We found that the expression of synaptophysin-ir puncta was not different in the NTS of naïve rats or rats with sham surgery (*p* > 0.05, unpaired t-test) (Fig. 2D-E, H). However, the expression of synaptophysin-ir puncta was significantly upregulated in rats with CMI compared with rats with sham surgery. Coincident with Fos expression, upregulation of synaptophysin-ir puncta mainly occurred in the SolM (sham, 2879.8 ± 153.8, CMI, 4579.8 ± 605.9; p = 0.03, n = 4 rats in each group, unpaired t-test) and SolC (sham, 3170.5 ± 427.8, CMI, 5257.5 ± 271.1; p = 0.006, n = 4 rats in each group, unpaired t-test) but not in the SolL (sham, 4205.3 ± 690.2, CMI, 3958.3 ± 824.0; p = 0.83, n = 4 rats in each group, unpaired t-test) (Fig. 2E-F, H). In total, our results indicate that left anterior descending coronary artery ligation can increase the expression of presynaptic terminals and activate local neurons in the SolM and SolC. Meanwhile, activation of the NTS was not observed in rats two weeks after sham surgery.

### Synaptophysin-ir terminals prefer to make a close connection to a Fos-ir neuron in the NTS

**Fig 3:**
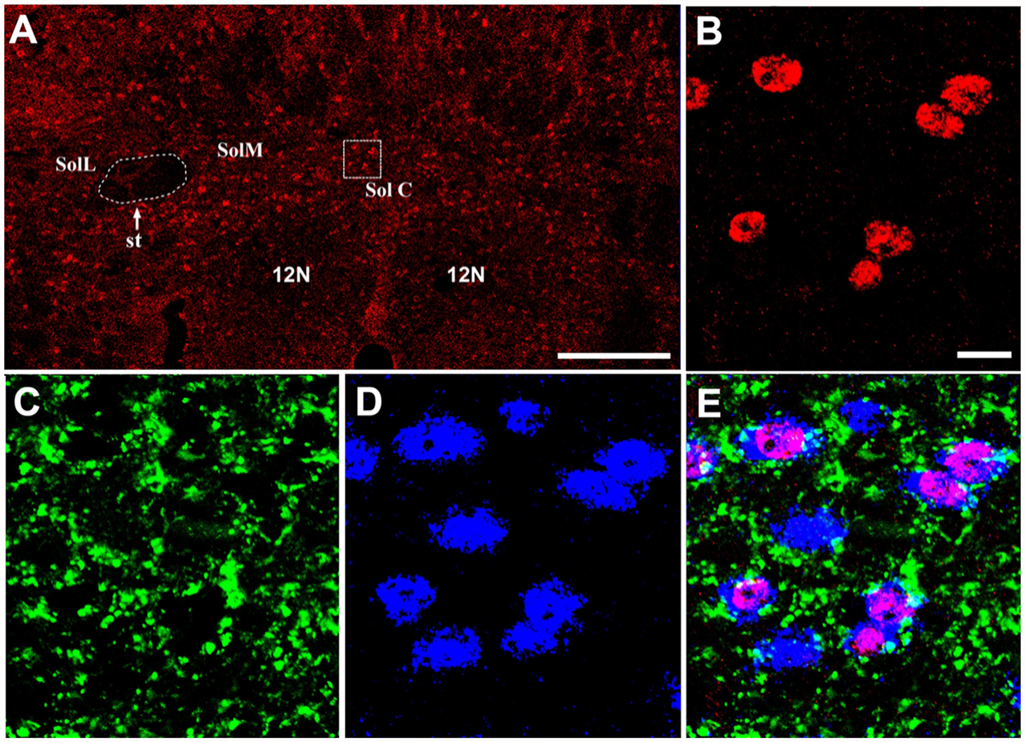
Fluorescent images showing that Fos-ir neurons were highly expressed in the NTS of rats with CMI. (A) A sample slice showing Fos-expression in the NTS. (B) A magnified image of the rectangle in (A). (C-D) The synaptophysin-ir terminals and NeuN-ir neurons shown within the same area as (B). (E) The merged image from (B-D) showing that synaptophysin-ir terminals made close connection to the NeuN-ir neurons, especially to those with Fos-immunoreactivity. 10n, dorsal motor nucleus of vagus; SolC, commissural part of the NTS; SolL, lateral part of the NTS; SolM, medial part of the NTS; st, solitary tract. Scale bars: 500 μm (A) and 20 μm (B-E).

NeuN (Hexaribonucleotide Binding Protein-3), a homologue of sex-determining genes in Caenorhabditis elegans, is commonly used as a biomarker for neurons. Under a confocal laser-scanning microscope, 23.3% of NeuN-ir neurons in the SolM and SolC expressed Fos protein in the sham group (NeuN: 824.8 ± 44.1; Fos: 190.8 ±16.7). After left anterior descending coronary artery ligation, the percentage of Fos-ir neurons increased to 40.6% (NeuN: 802.3 ± 39.3; Fos: 325.3 ± 38.4). The percentage of NeuN-ir neurons expressing Fos was significantly different between the sham and CMI groups (p = 0.017, n = 4 rats in each group, unpaired t-test). By exploring triple-immunostaining for Fos, NeuN and synaptophysin in rats with CMI, we showed that synaptophysin-ir terminals make close connection to NeuN-ir neurons in the SolM and SolC, with a preference for those with Fos-immunoreactivity, suggesting that neuronal activation (with Fos expression) may be induced by the enhanced input in rats with CMI (Fig. 3).

### CMI induced potentiation of excitatory pre- and postsynaptic transmission

Morphological work indicated that CMI increased the numbers of presynaptic terminals and activated postsynaptic neurons in the medial and commissural part of the NTS. We thus used the whole-cell patch clamping method to test whether synaptic transmission efficiency in the NTS was different between rats with CMI or sham surgery.

**Fig 4:**
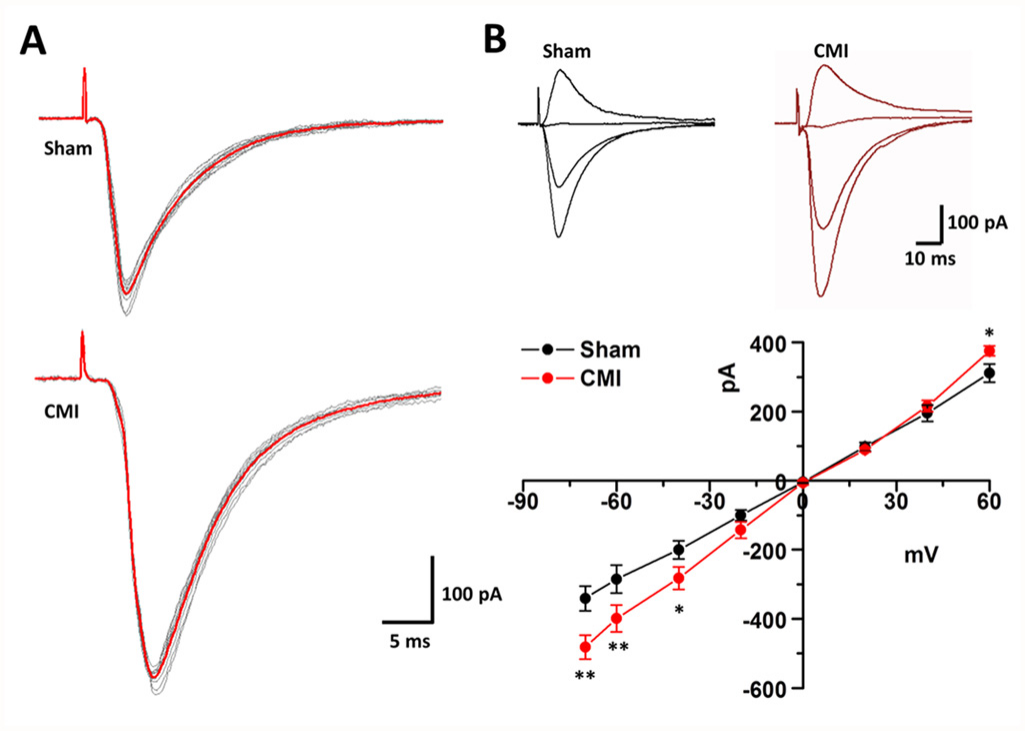
CMI potentiated tractus solitaries stimulation-evoked monosynaptic responses in the SolM. (A) Representative sample traces showing the single (gray) and averaged (red) monosynaptic responses induced by tractus solitaries stimulation in rats with sham surgery (up) or CMI (down). The monosynaptic nature was clarified by the constant latencies and small jitter. (B) Inset samples showing the superimposed monosynaptic responses at various holding potentials (-70, -40, 0 and +40 mV) in rats with sham surgery or CMI. The plotted figure shows the averaged I–V curve of the evoked responses, indicating that there was a significant inward rectification of the I-V curve in rats with CMI. *, *p* < 0.05; **, *p* < 0.001 compared with the sham group.

We first examined the AMPAR-mediated EPSC in SolM neurons elicited by stimulating the tractus solitarius (TS). Only those neurons that received direct inputs from the TS were analyzed. In accordance with previous reports [13,19,20,21], the evoked EPSCs (eEPSCs) were monosynaptic, with obvious but short latencies (sham: 3.36 ± 0.31 ms, CMI: 3.44 ± 0.34 ms, *p* = 0.86, n = 25 in sham and 28 in CMI group, unpaired t-test) and small jitter from the onset of the stimuli (<180 μs) in rats with either CMI or the sham operation (Fig. 4A). Meanwhile, increasing the stimuli intensity did not increase eEPSC amplitude. In rats with the sham operation, the amplitude of the eEPSC was 344.7 ± 22.1 pA (n = 29). In rats with CMI, however, the amplitude was significantly increased (446.8 ± 23.0 pA, n = 31. *p* < 0.001, unpaired t-test), indicating that CMI increases the efficacy of synaptic transmission in the NTS (Fig. 4A). The AMPAR-mediated EPSCs at different holding potentials (-70 to +60 mV) were also recorded. We found that in rats with CMI, there was an obvious inward rectification of the mean I-V curve (*F*_(1, 112)_ = 11.89, *p* < 0.001, n = 8 in each group, two-way ANOVA followed by *post hoc* comparison with LSD test). The rectification index decreased significantly (sham surgery: 0.95 ± 0.04, CMI: 0.73 ± 0.08; n = 8 in each group; *p* = 0.02, unpaired t-test) (Fig. 4B).

**Fig 5:**
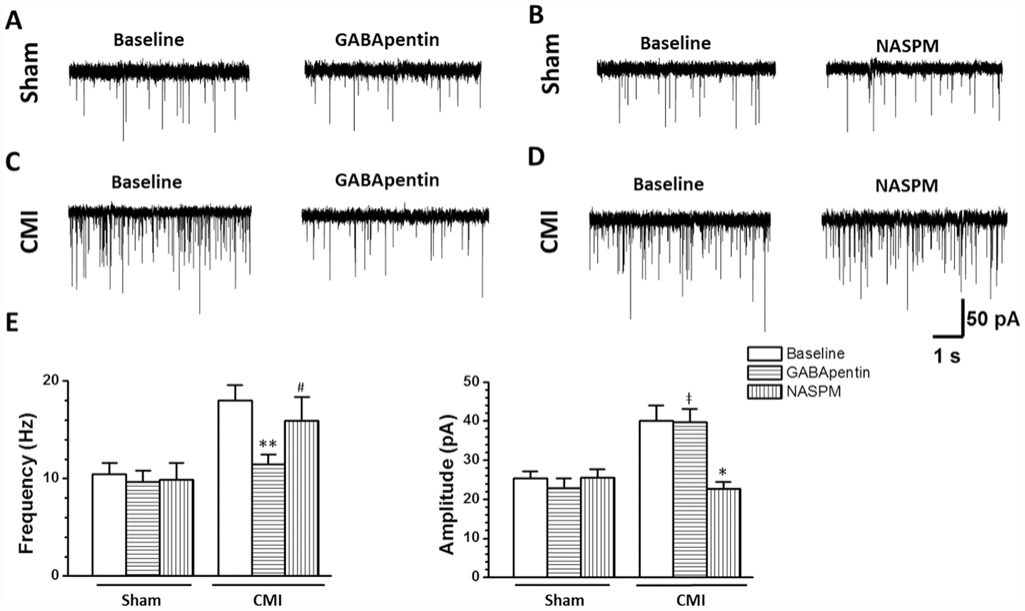
GABApentin and NASPM respectively inhibited the frequency and amplitude of the mEPSC in the NTS. (A-B) Bath application of GABApentin (50 μm) or NASPM (50 μM) had no effect on the frequency and amplitude of the mEPSC in rats with sham surgery. (C) GABApentin inhibited the frequency but not the amplitude of the mEPSC in rats with CMI. (D) NASPM decreased the amplitude but not the frequency of the mEPSC in CMI rats. (E) Summarized results for the effects of GABApentin and NASPM on the mEPSC in rats with sham or CMI treatment. #, NASPM vs. baseline, *p* > 0.05; ǂ, GABApentin vs. baseline, *p* > 0.05; *, NASPM vs. baseline, *p* < 0.05; **, GABApentin *vs*. baseline, *p* < 0.01.

**Fig 6:**
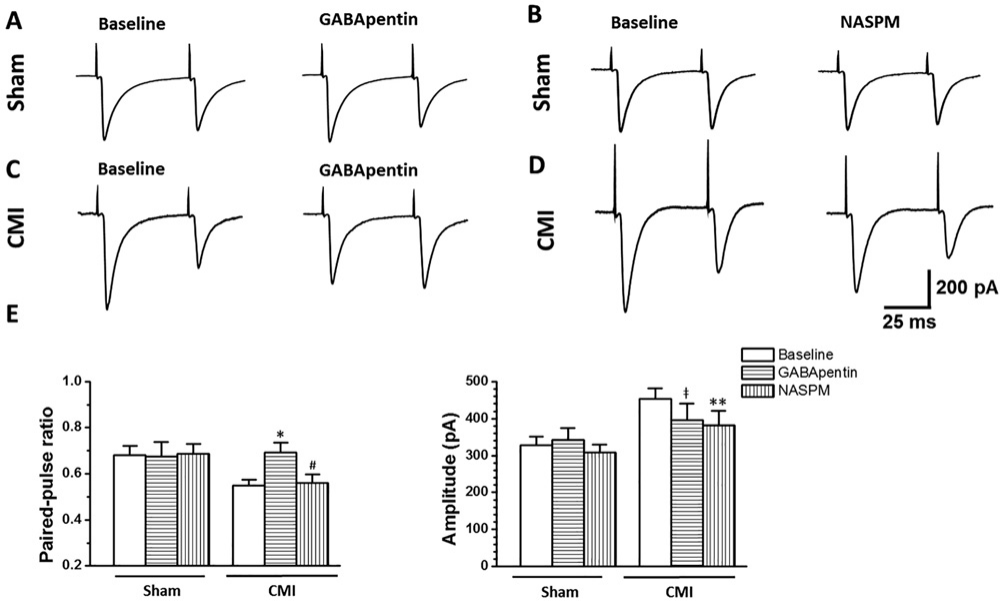
The paired-pulse responses were affected by GABApentin and NASPM in different ways. (A-B) Bath application of GABApentin (50 μm) or NASPM (50 μM) has no effect on the ratio and amplitude of the paired-pulse depression (PPD) in rats with sham surgery. (C) GABApentin increased the ratio of the PPD and inhibited the amplitude of the first eEPSC in rats with CMI. (D) NASPM decreased the amplitude of the paired responses but did not affect the paired-pulse ratio in CMI rats. (F) Summarized results for the effects of GABApentin and NASPM on the paired-pulse responses in rats with sham or CMI treatment. #, NASPM vs. baseline, *p* > 0.05; ǂ, GABApentin vs. baseline, *p* > 0.05; *, GABApentin vs. baseline, *p* < 0.05; **, NASPM *vs*. baseline, *p* < 0.01.

Miniature excitatory post-synaptic current (mEPSC) and paired-pulse ratio (PPR), two simple measurements for analyzing presynaptic transmitter release probability and postsynaptic responses, were then tested. The results showed that, compared with the sham group, the frequency and amplitude of the mEPSC in rats with CMI increased significantly (frequency: sham, 10.5 ± 1.1 Hz, CMI, 18.0 ± 1.6 Hz, *p* < 0.001; amplitude: 25.3 ± 1.8 pA, CMI, 40.1 ± 3.8 pA, *p* = 0.001. n = 17 neurons in each group, unpaired t-test) (Fig. 5). When applying paired-pulse stimuli, we found that most of the recorded neurons exhibited paired-pulse depression (PPD), which was in accordance with previous reports [13,19,21]. In rats with CMI, PPR was decreased (sham, 0.68 ± 0.04, CMI, 0.55 ± 0.03, n = 19 neurons in the sham group and 23 neurons in the CMI group, *p* = 0.008, unpaired t-test). However, the amplitude of the first eEPSC was potentiated (sham, 328.0 ± 23.3 pA, CMI, 456.7 ± 27.9, *p* = 0.001, unpaired t-test) (Fig. 6). In total, these results indicated that CMI potentiated presynaptic glutamate release and postsynaptic AMPA receptor-mediated responses.

### GABApentin inhibited the enhanced presynaptic transmitter release probability in rats with CMI

GABApentin is a widely used antiepileptic and antihyperalgesic agent, which has an inhibitory effect on the activity of presynaptic P/Q and N type Ca^2+^ channels, and thus inhibits presynaptic transmitter release in the spinal cord and brainstem [13,22,23]. We thus used GABApentin to check whether the enhanced probability of presynaptic transmitter release could be reversed by GABApentin (recorded baseline for 20 min and then bath applied GABApentin for another 20 min). We found that the frequency (baseline, 19.8 ± 2.1 Hz; GABApentin, 11.5 ± 1.0 Hz, n = 8 neurons, *p* = 0.01, paired t-test) but not the amplitude (baseline, 46.4 ± 4.3 pA; GABApentin, 39.9 ± 3.4 pA, n = 8 neurons, *p* = 0.10, paired t-test) of the mEPSC was inhibited by GABApentin (50 μM) application in rats with CMI but not in rats with sham surgery (frequency: baseline, 9.0 ± 1.3 Hz, GABApentin, 9.8 ± 1.1 Hz, *p* = 0.36; amplitude: baseline, 26.7 ± 1.9 pA; GABApentin, 24.3 ± 2.6 pA, *p* = 0.14. n = 9 neurons, paired t-test) (Fig. 5A, C, E). Similarly, application of GABApentin increased the ratio of PPD in rats with CMI (baseline, 0.57 ± 0.04; GABApentin, 0.69 ± 0.04, *p* = 0.03. n = 12 neurons, paired t-test) but not in rats with sham surgery (baseline, 0.70 ± 0.06; GABApentin, 0.67 ± 0.06, *p* = 0.57. n = 10 neurons, paired t-test) (Fig. 6A, C, E). These results indicated that GABApentin inhibited presynaptic glutamate transmission in rats with CMI.

### NASPM inhibited the potentiated postsynaptic responses in rats with CMI

AMPAR is a heterotetramer of four homologous subunits (GluR1 to GluR4) that combine in different stoichiometries to form different subunits [24]. In normal conditions, most of the AMPARs contain the GluR2 subunit. During plastic synaptic changes, GluR2 can be replaced by the GluR1/3 subunit and can cause potentiated post-synaptic responses [25,26,27,28]. Because GluR1/3 is inwardly rectifying, we expected that the potentiated AMPAR-mediated responses may have been due to increased expression of the Ca^2+^ permeable GluR1/3 subunit, based on the inward rectification of the mean I-V curve in rats with CMI (Fig. 4B). We thus bath applied the GluR1/3 subunit antagonist 1-Naphthylacetyl spermine trihydrochloride (NASPM) to check whether NASPM can inhibit the CMI-induced potentiation of the postsynaptic responses. After bath application of NASPM (50 μM), the amplitude (baseline, 34.6 ± 5.7 pA; NASPM, 22.7 ± 1.9 pA, n = 9 neurons, *p* = 0.02, paired t-test) but not the frequency (baseline, 16.5 ± 2.3 Hz; NASPM, 15.9 ± 2.5 Hz, n = 9 neurons, *p* = 0.37, paired t-test) of the mEPSC was greatly reduced in rats with CMI but not in rats with sham surgery (frequency: baseline, 12.1 ± 1.8 Hz, NASPM, 9.9 ± 1.7 Hz, *p* = 0.14; amplitude: baseline, 23.8 ± 3.2 pA; NASPM, 25.6 ± 2.2 pA, *p* = 0.44. n = 8 neurons, paired t-test) (Fig. 5B, D, E). Similarly, NASPM did not change the PPD ratio (baseline, 0.53 ± 0.04; NAS308.PM, 0.56 ± 0.04, n = 11 neurons, *p* = 0.56, paired t-test) but decreased the amplitude of the first eEPSC (baseline, 453.6 ± 43.5 pA; NASPM, 381.7 ± 38.9 pA, n = 11 neurons, *p* = 0.005, paired t-test) (Fig. 6D, E) in rats with CMI. Meanwhile, NASPM had no effect on the PPR (baseline, 0.66 ± 0.05; NASPM, 0.69 ± 0.04, n = 9 neurons, *p* = 0.44, paired t-test) and the amplitude of the first eEPSC (baseline, 296.4 ± 25.6 pA; NASPM, 308.3 ± 21.45 pA, n = 9 neurons, *p* = 0.19, paired t-test) in rats with sham surgery (Fig. 6B, E). These results indicated that, unlike GABApentin, NASPM inhibited postsynaptic responses mediated by AMPA receptors.

### Microinjection of NASPM or GABApentin into the NTS reversed CMI-induced visceral pain behavior

**Fig 7:**
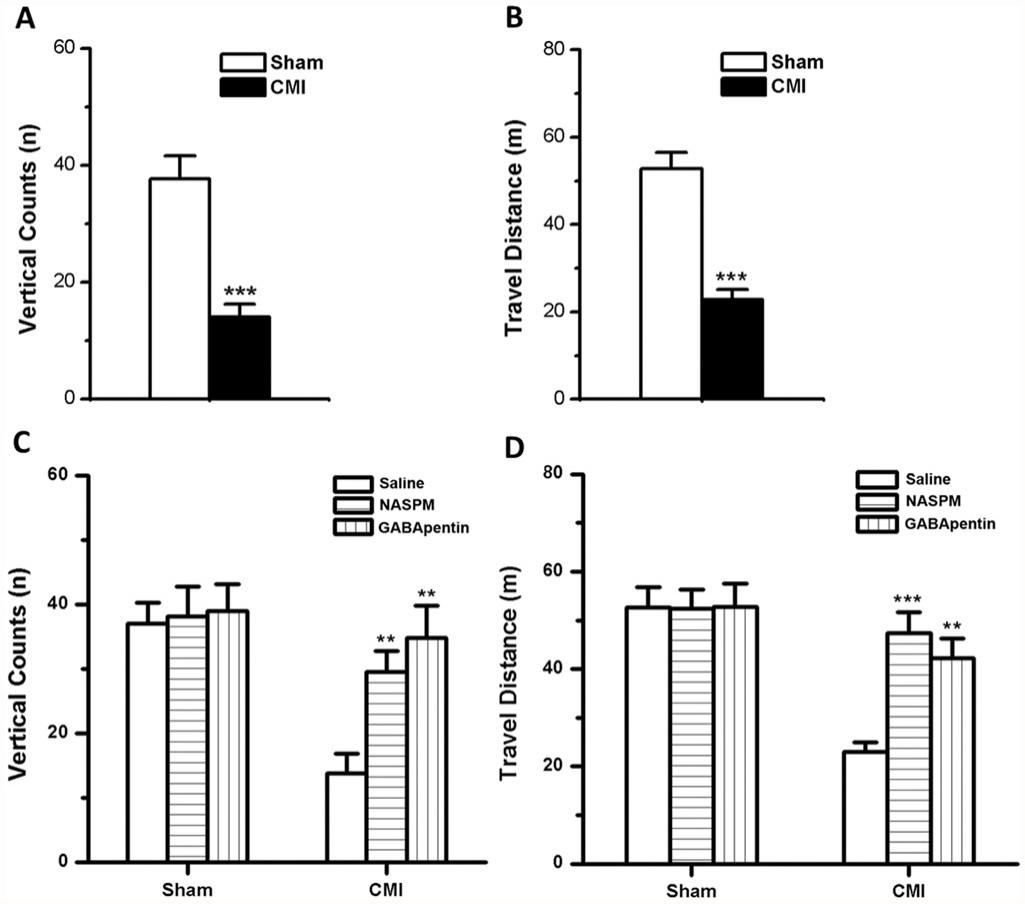
Behavioral assessment of rats in the open field. (A-B) Vertical counts (A) and travel distance (B) were decreased in rats with CMI. ***, *p* < 0.001 compared with the sham group. (C-D) Microinjection of NASPM or GABApentin into the SolC significantly increased the vertical counts (C) and travel distance (D) in CMI rats. **, *p* < 0.01; ***, *p* < 0.001 compared with the CMI group with saline microinjection.

We also evaluated the vertical (rearing) counts and travel distance using the open field test. In our previous work, we confirmed that these items can be used to evaluate visceral pain [17]. In the present study, we expect they reflect the severity of CMI-induced angina pectoris. To avoid habituation effects, independent groups were used on each testing day. Thus, each rat was tested only once in the open field. The vertical counts were significantly decreased in CMI rats compared with the sham group (Sham: 37.7 ± 4.0, CMI: 14.0 ± 2.2; *p* < 0.001, n = 6 rats in each group, unpaired t-test. Fig. 7A). The travel distance of CMI rats in the open field also decreased markedly (Sham: 52.8 ± 3.7 m, CMI: 22.8 ± 2.4 m; *p* < 0.001, n = 6 rats in each group, unpaired t-test. Fig. 7B). These results consistently suggest that CMI rats experience more severe visceral pain than rats in the sham group.

We then microinjected saline, NASPM or GABApentin into the NTS to test their effects on the vertical counts and travel distance. Drugs were microinjected into the SolC 45 min prior to the open field test. In comparison with the saline group (vertical counts: 13.8 ± 3.0; travel distance: 23.0 ± 2.0 m), microinjection of NASPM or GABApentin increased both the vertical counts (NASPM: 29.5 ± 3.3, *p* = 0.009; GABApentin: 34.8 ± 5.0, *p* = 0.001) and the travel distance (NASPM: 47.4 ± 4.4 m, *p* < 0.001; GABApentin: 42.2 ± 4.0 m, *p* = 0.002) in rats with CMI (n = 6 rats per group, one-way ANOVA followed by *post hoc* comparison with LSD test) (Fig. 7C-D). Meanwhile, microinjection of saline, NASPM or GABApentin had no effect on the vertical counts and travel distance in rats with sham surgery (*p* > 0.05. n = 6 rats per group, one-way ANOVA) (Fig. 7C-D). These results suggest that NASPM and GABApentin can alleviate CMI-induced visceral pain in rats.

### Baroreceptor responses were not changed two weeks after coronary artery ligation surgery

In addition to modulating cardiac nociceptive information, the NTS is critical for integrating and regulating sensory information from peripheral baroreceptors [29,30]. Therefore, we cannot exclude the possibility that the morphological and electrophysiological changes in the NTS and the increased visceral pain behavioral responses are affected by cardio-respiratory responses. We thus measured the heart rate (HR), mean arterial pressure (MAP) and breath ratio (BR) in rats two weeks after coronary artery ligation surgery. We found that HR, MAP and BR were not different between the CMI, sham and naïve groups (Table 1). Thus, the response changes in the NTS and the increased visceral pain behavior may mainly come from CMI-induced angina pectoris and are less likely to be affected by the cardio-respiratory reflex in rats weeks after coronary artery ligation surgery.

**Table 1:** Hemodynamic measurements and breathing rate in naïve, sham-operated and CMI rats.

	Naïve (n = 6)	Sham (n = 6)	CMI (n = 6)	*F*_(2, 15)_	*P*
HR (bpm)	345 ± 29	355 ± 37	363 ± 30	0.08	0.92
MAP (mm Hg)	90 ± 6	89 ± 9	85 ± 5	0.59	0.57
BR (fpm)	91 ± 6	90 ± 7	96 ± 5	0.32	0.73

HR, heart rate; bpm, beats per minute; MAP, mean arterial pressure; BR, breathing rate; fpm, frequency per minute. One-way ANOVA.

## Discussion

In the present study, by exploring left anterior descending coronary artery ligation surgery, we studied the morphological and functional changes in the NTS of rats with CMI. The overexpression of synaptophysin- and Fos-immunoreactivity and the potentiated excitatory pre- and postsynaptic transmission strongly indicate that NTS sensitization may be significantly involved in angina pectoris caused by chronic occlusion of the coronary artery.

Angina is traditionally considered a consequence of the supply/demand mismatch caused by the undersupply of myocardial oxygen. Thus, classic therapeutic approaches mainly aim to surgically vascularize the heart, pharmacologically increase myocardial blood supply or reduce cardiac oxygen consumption [2,31,32]. However, in many cases, clinical therapy cannot reach a satisfactory outcome [33], especially in patients with cardiac syndrome X or intractable angina pectoris, in which the coronary arteries are usually less damaged, although severe angina occurs frequently and have a long duration [2]. Central sensitization on the spinal and supraspinal levels may contribute to the complicated cardiac symptoms because central sensitization commonly regulates the intensity of somatic nociception [32]. This proposal is strongly supported by the finding that spinal sympathectomy significantly reduces the frequency of angina attacks in patients with intractable angina [34] and is further confirmed by a similar treatment effect observed in patients with cardiac syndrome X [35,36]. However, although brain structures, such as the NTS, parabrachial area, cingulate cortex, etc., have all been proposed as important structures for the regulation of cardiac nociceptive information, related studies are rather scarce. In our previous work, we found that the NTS is important for regulating the intensity of the acute cardiac-somatic reflex. Furthermore, microinjections of NMDA receptors and group III mGluRs antagonists into the NTS have different regulatory effects (Liu et al., 2012a, Liu et al., 2012b). The present results deepen our understanding of the function of NTS in the modulation of cardiac nociceptive information and, for the first time, show that inhibiting potentiated excitatory synaptic transmission would be beneficial for treating angina pectoris.

Glutamate is the main excitatory transmitter for intercellular information transport. Bath application of GABApentin, which inhibited the activity of presynaptic Ca^2+^ channels and reduced the release of glutamate from axon terminals, and NASPM, which inhibited the activity of postsynaptic Ca^2+^ permeable GluR1/3, could separately reverse the potentiated pre- and postsynaptic transmission in rats with CMI. These results provide a new strategy for angina treatment and show that the pre- and postsynaptic influx of Ca^2+^ is important for CMI-induced synaptic potentiation. One interesting finding was that the Ca^2+^ permeable AMPAR receptor subunits (GluR1/3) replaced the GluR2 in the synaptic region after coronary artery ligation. It is already well documented that the NMDA receptor is important for triggering synaptic plasticity induced by various stimuli, such as learning, fear and pain induction [37,38,39,40,41] and for triggering the postsynaptic accumulation of GluR1 and the replacement of GluR2 with GluR1/3 [24,26]. Thus, it is likely that the potentiated postsynaptic response is initiated by overexcited NMDAR and the mediated by subsequent trafficking of GluR1/3 in the PSD. Actually, in our pilot work, we have found that NMDAR subunit NR2A and NR2B were overexpressed in the NTS with CMI (unpublished data). Tang et al. also report that tetanic stimulation of the aortic depressor nerve potentiated the A-fiber evoked responses in the NTS, which can be blocked by NMDA receptor antagonists [42], indicating the role of NMDAR in the potentiation of synaptic transmission. However, more works should be carried out in the future to clarify the role of NMDAR for the synaptic plasticity in the NTS.

Another interesting finding is that the increased expression of synaptophysin-ir puncta and Fos-ir neurons mainly occurred in the SolM and SolC. Because the SolM and SolC but not SolL are the main targets for afferent vagal fibers [43], it is likely that nociceptive information from the heart will increase the amount of afferent vagal terminals and then activate the neurons within the SolM and SolC. Actually, our previous work showed that microinjection of the NMDA receptor antagonist MK801 into the SolC but not the SolL inhibits the acute cardiac-somatic reflex (Liu et al., 2012b). However, we cannot rule out that the increased afferent terminals may also come from the ascending fibers of the spinal cord. To address this question, selective vagotomy or sympathectomy should be explored in the future.

### Conclusions

In the present study, by combining morphological and functional methods, we found that the NTS is involved in the central modulation of angina pectoris after left anterior descending coronary artery ligation in adult rats. Inhibition of the potentiated excitatory pre- and postsynaptic transmission in the NTS will provide potential value for the treatment of angina pectoris resulting from chronic myocardial infarction.
